# Genotypic Frequencies of Mutations Associated with Alpha-1 Antitrypsin Deficiency in Unrelated Bone Marrow Donors from the Murcia Region Donor Registry in the Southeast of Spain

**DOI:** 10.3390/diagnostics13172845

**Published:** 2023-09-02

**Authors:** Irene Cuenca, Carmen Botella, María Rosa Moya-Quiles, Víctor Jimenez-Coll, José Antonio Galian, Helios Martinez-Banaclocha, Manuel Muro-Pérez, Alfredo Minguela, Isabel Legaz, Manuel Muro

**Affiliations:** 1 Immunology Service, University Clinical Hospital “Virgen de la Arrixaca”, Biomedical Research Institute of Murcia (IMIB), 30120 Murcia, Spain; 2 Department of Legal and Forensic Medicine, Biomedical Research Institute of Murcia (IMIB), Regional Campus of International Excellence “Campus Mare Nostrum”, Faculty of Medicine, University of Murcia (UMU), 30100 Murcia, Spain

**Keywords:** AAT1 deficiency, chronic obstructive pulmonary disease, SERPINE1 gene, Pi system

## Abstract

Alpha-1 antitrypsin (AAT1) deficiency (AAT1D) is an inherited disease with an increased risk of chronic obstructive pulmonary disease (COPD), liver disease, and skin and blood vessel problems. AAT1D is caused by mutations in the SERPINE1 gene (Serine Protease Inhibitor, group A, member 1). Numerous variants of this gene, the Pi system, have been identified. The most frequent allelic variants are Pi*M, Pi*S, and Pi*Z. The development of COPD requires both a genetic predisposition and the contribution of an environmental factor, smoking being the most important. Studies on this deficiency worldwide are very scarce, and it is currently considered a rare disease because it is underdiagnosed. The aim of this study was to analyze the genotypic frequencies of mutations associated with AAT1 deficiency in unrelated bone marrow donors from the donor registry of the Region of Murcia in southeastern Spain due to the high risk of presenting with different pathologies and underdiagnosis in the population. A total of 112 DNA-healthy voluntary unrelated bone marrow donors from different parts of the Region of Murcia were analyzed retrospectively. AAT1 deficiency patient testing involved an automated biochemical screening routine. The three main variants, Pi*M, Pi*Z, and Pi*S, were analyzed in the SERPINE1 gene. Our results showed a frequency of 3.12% of the Pi*Z (K342) mutation in over 224 alleles tested in the healthy population. The frequency of Pi*S (V264) was 11.1%. The frequency of the haplotype with the most dangerous mutation, EK342 EE264, was 4.46%, and the frequency of EK342 EV264 was 1.78% in the healthy population. Frequencies of other EE342 EV264-mutated haplotypes accounted for 18.7%. As for the EE342 VV264 haplotype, 0.89% of the total healthy population presented heterozygous for the EV264 mutation and one individual presented homozygous for the VV264 mutation. In conclusion, the frequencies of Pi mutations in the healthy population of the Region of Murcia were not remarkably different from the few studies reported in Spain. The genotype and haplotype frequencies followed the usual pattern. Health authorities should be aware of this high prevalence of the Pi*S allelic variant and pathological genotypes such as Pi*MZ and Pi*SZ in the healthy population if they consider screening the smoking population.

## 1. Introduction

Alpha-1 antitrypsin (AAT1) is a water-soluble serum glycoprotein characterized by circulating in peripheral blood and diffusing in tissues [1] that inhibits the activity of proteolytic enzymes, the primary target of which is neutrophil elastase (NE), particularly in the lower respiratory tract [1]. AAT1 has a relevant anti-oxidant capacity that protects tissues from oxidative damage [2] and multiple anti-inflammatory and tissue-protective properties independently of its anti-protease activity. Similarly, AAT1 has an antimicrobial property as it can inhibit the replication and infectivity of certain viruses, such as HIV, bacteria, and protozoa, such as Cryptosporidium parvum [3,4]. AAT1 inhibits and reacts with NE with an association constant 25 times higher than that applied to neutralize other serine proteases such as trypsin, proteinase-3, alpha-defensins, cathepsin G, and plasminogen activator [5,6,7]. Other studies indicate that the inactivation of matriptase carried out by AAT1 could mediate sodium transport and promote mucociliary clearance in people with COPD and cystic fibrosis [8,9].

AAT1 deficiency (AAT1D) is caused by mutations in the SERPINE1 gene (Serine Protease Inhibitor group A, member 1) [10], located on the long arm of chromosome 14 (14q32.1). Some mutations in the SERPINE1 gene result in either little or no AAT1 to protect the lungs or its abnormal accumulation in the liver [11]. The disease is inherited in a codominant autosomal fashion through two alleles, manifesting independently in 50% of the children [12]. AAT1D is an inherited disease in which there is an increased risk of chronic obstructive pulmonary disease (COPD) [13,14,15], liver disease, and also skin (panniculitis) and blood vessel (vasculitis) problems [16,17,18]. AAT1D results in an elevation of NE activity in the lung which causes damage to the alveolar wall [18,19]. The resulting COPD is the most prevalent clinical manifestation of AAT1D, although symptoms of liver abnormalities may also present in childhood. In non-smoking individuals with AAT1D, the first symptoms of lung disease occur at a mean age of 45; however, it is reduced to 35 years of age in smokers [11,20]. AAT1D smokers show a significantly higher rate of lung destruction and have a worse survival rate than AAT1D non-smokers [21].

Numerous variants of the AAT1 gene have been identified, which is why the set of AAT1 electrophoretic variants is called the Pi (protease inhibitor) system [12]. The variants are classified according to their electrophoretic migration speed in isoelectric focusing gel; for this reason, those with medium migration speed are called Pi M (M for medium), those with slow migration speed are called Pi S (S for slow), those with fast migration speed are called Pi F (F for fast), and those with very slow migration speed are called Pi Z [22,23]. The mutant in the Z allele causes an amino acid change (glutamic acid at position 342 to lysine (E342K)), which alters the protein’s functionality leading to a structural modification that induces polymerization. The protein is less functional once secreted. In contrast, the mutant in the S allele causes a change from glutamic acid to valine at position 264 (E264V) [23].

The treatment of AAT1D is based on controlling the symptoms and includes using bronchodilators, corticosteroids, antibiotics, and oxygen, if the patient suffers from advanced respiratory failure. However, there is currently no cure. Depending on the patients’ different clinical pictures, patients presenting with pulmonary emphysema with severe AAT1D (AAT1D concentrations equal to or less than 50 mg/dL) will be treated with replacement therapy with purified AAT1 obtained from donor plasma and administered intravenously [24], and, in more severe cases, may require a lung or liver transplant [3].

The aim of this study was to analyze the genotypic frequencies of mutations associated with AAT1 deficiency in unrelated bone marrow donors from the donor registry of the Region of Murcia in southeastern Spain due to the high risk of presenting with different pathologies and underdiagnosis in the population.

## 2. Materials and Methods

### 2.1. Demographic Information, Clinical Variables, and Study Methodology

A total of 112 healthy voluntary unrelated bone marrow donors from different parts of the Region of Murcia were evaluated at the University Clinic Hospital “Virgen de la Arrixaca (HCUVA)” (Spain) and retrospectively analyzed. The inclusion criteria for this study were acceptance of informed consent by the donor, available DNA sample at an adequate concentration and purity, and knowledge of no individual or family history of previous lung disease.

**Figure 1:**
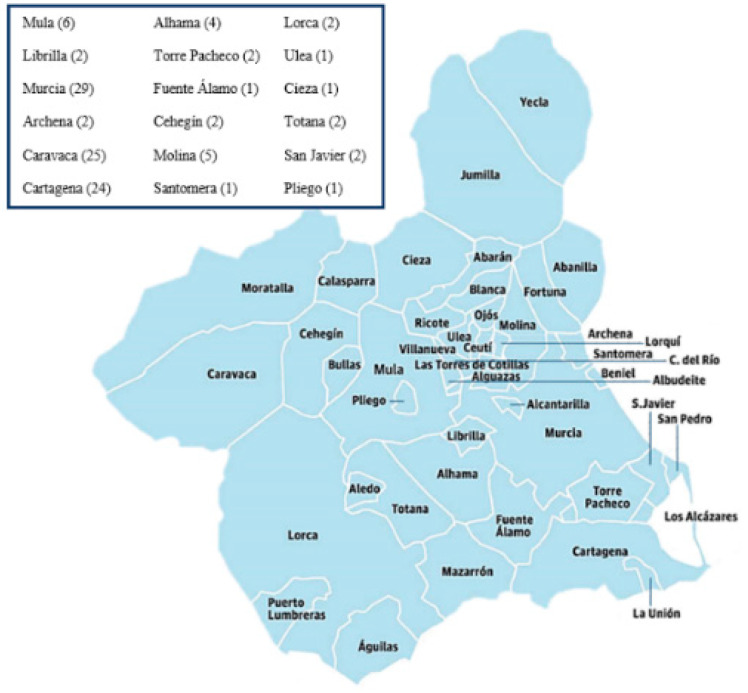
Map of the Region of Murcia showing the origin and number of study subjects from different municipalities (Source: Laboratory Information System (SIL) of the Immunology Service of the HCUVA, Modulab of the Werfen company, Barcelona, Spain).

EDTA samples were taken where genomic DNA had been extracted from all the individuals included in the study (chosen at random) with the guarantee that they did not present family ties to other donors. Of these bone marrow donors, 35% (39) were men, and 65% (73) were women, with a mean age of 29.0 years ± 3.2; mean ± SD. The samples used in this study are representative of the Murcian population as they include individuals from most of the region’s municipalities (Figure 1).

Written informed consent of all participants was obtained before the start of this clinical investigation. In addition, the protocols of this clinical study were approved by the HCUVA Clinical Research Ethics Committee (PI15/01370). The clinical study was carried out according to the agreement of the Declaration of Helsinki 2000 [25].

### 2.2. Genomic Extraction

Genomic DNA was extracted from human peripheral blood using the QIASymphony DNA Mini Kit (Izasa, Werfen, US). All extracted samples were quantified by Nanodrop technology (Promega, Madison, WI, US). The study did not include any samples below 1.5 of the A260/280 ratio. Finally, all the extracted and quantified samples were stored at −20 °C until later use.

### 2.3. PCR Amplification-Refractory Mutation System (ARMS-PCR)

AAT1 deficiency testing has previously and classically involved an automated biochemical screening routine in identifying patients with low serum levels of the AAT1 glycoprotein (Phadia^TM^, Thermo Fisher Scientific, Carlsbad, CA, USA); this is the classical, usual procedure in samples of patients with suspicions of AAT1 deficiency. In the case of altered AAT1 values, classically the procedure is followed by isoelectric focusing (IEF), a technique for separating and identifying protein variants. Many variants of the SERPINE1 gene have been identified, but relatively few cause severe AAT1 deficiency.

This study analyzed the wild-type alleles (Pi*M) and the variants Pi*Z and Pi*S (Table 1). The mutant Pi*Z allele causes the change of glutamic acid to lysine at position 342 (E342K), while the mutant Pi*S allele causes the change of glutamic acid to valine at position 264 (E264V). Pi*Z and Pi*S variants are caused by point mutations in the SERPINE1 gene and are associated with reduced plasma AAT1 levels and abnormal functioning of the Z protein [26].

The ARMS–PCR amplification technique is an allele-specific PCR amplification technology capable of detecting point mutations or deletions in DNA. Applying this analysis to samples of healthy patients makes it possible to identify the carriers of the deficient genes since, in some cases, these people have normal serum levels of AAT1 (85–210 mg/dl). The kits used were Elucigene AAT Kit (ref. AA002B2, Delta Diagnostics, Manchester, UK)). Each kit consists of two reaction mixes. One amplifies the normal or wild-type allele explicitly, that is, all the alleles unaffected by the S or Z mutations, and the other contains primers that specifically amplify only the mutant S and Z alleles. Likewise, each reaction mixture includes primers that amplify sequences of DNA outside the AAT1 gene that serve as an internal control for amplification.

Briefly, the AmpliTaq Gold was activated at 94 °C (20 min) and linked to a PCR (35 cycles), developed in a thermocycler (Applied Biosystems, Thermo Fisher, Carlsbad, CA, USA) and consisting of the following thermocycling parameters: denaturation at 94 °C (30 s), alignment at 62 °C (1 min), extension of 72 °C (1 min), and final extension of 72 °C (20 min). The total time was approximately 3 h. Subsequently, the PCR products were run on a 3% agarose gel. The DNA ladder ΦX174 DNA/HaeIII (Promega) ranging in size from 72 bp to 1353 bp was used. Next, 15 µL of the PCR product was added to each well of the gel. Electrophoresis was then carried out at 80–90 V until the stained front had migrated towards the anode about 4 cm from the loading wells. This process usually took approximately 90 min. Finally, after completing the electrophoresis, the gel was read in a UV-transilluminator at 260 nm and photographed.

### 2.4. Statistical Analysis

The results were expressed as percentages for categorical data. A χ^2^ test or Fisher’s exact test were used to compare categorical variables. The verification of the normality of the data was carried out using the Kolmogorov–Smirnov test, as previously published [29]. The graphs and statistical analyses were performed using SPSS software (version 22, Chicago, IL, USA) and GraphPad Prism (version 6, San Diego, CA, USA) [30]. The *p*-value was corrected in multiple comparisons using the Benjamini–Hochberg or Bonferroni methods. For all statistical tests, *p* < 0.05 (or p-corrected 0.05 in the event of multiple comparisons) was considered significant [27].

## 3. Results

### 3.1. AAT1 Genotype Analysis

**Figure 2:**
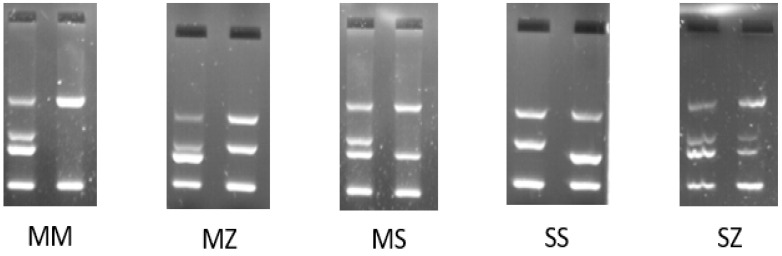
The most representative results for Pi* genotypes were obtained for the following mutations: MM, MZ, MS, SS, or SZ.

After the corresponding amplifications and the running of the PCR products on agarose gel, possible Pi* genotype models were obtained (Figure 2). The Pi*MM genotype revealed two wild-type alleles. Heterozygous Pi*MZ, *MS, and Pi*SZ genotypes indicated the presence of one wild-type allele and one mutated allele. Analogously, when homozygous Pi*SS or Pi*ZZ genotypes were obtained, the individual presented with two identical mutated alleles.

### 3.2. Analysis of Alleles of the Pi*Z and Pi*S Variants in the SERPINE1 Gene

The allele frequencies of the SERPINE 1 gene are shown in Table 2 (upper section). A frequency of 3.12% was observed for the codon that codes for glutamic acid, giving rise to Lysine (K^342^) in over 224 alleles tested in the healthy population. Our results showed that seven healthy individuals (3.12%) presented with the mutated allele K^342^ (mutant) with implications for an eventual precise genetic diagnosis. The frequency of the other mutated allele, which codes for V264, stood at 11.1%; since this allele presents less clinical or pathological relevance, conducting genetic counseling would not be necessary.

### 3.3. Analysis of Genotype Frequencies of Pi*Z (E342K) and Pi*S (E264V) AAT1 Mutations

Table 2 (lower section) shows the genotypic frequencies of AAT1 mutations, detecting the percentage of genotypes that carried the EK342 variation at 6.25% in the healthy population; that is, seven healthy individuals presented with this mutated genotype with implications for an eventual precise genetic diagnosis since, as previously mentioned, no KK342 variations were observed in any of the cases.

As for the frequency of the **EV264** variation, 20.5% of the healthy population presented heterozygous for the mutation and one individual presented homozygous for said VV264 mutation. Since this allele presents less clinical relevance, conducting genetic counseling would not be necessary.

### 3.4. Analysis of Haplotype Frequencies of E342K and E264V in the SERPINE1 Gene

Table 3 shows the haplotype frequencies of the E342 and E264V AAT1 mutations (upper section), detecting the frequency of the haplotype that carries the most dangerous mutation, EK342 EE264, at 4.46%, and that of EK342 EV264 at 1.78% in the healthy population; that is, seven healthy individuals from our region presented with the haplotype that carries this mutation combined with the E264V variants, with implications for an eventual precise genetic diagnosis, since if these individuals were parents they would transmit this mutated allele with a probability of 50% and could have offspring that would become carriers and could even be homozygous (if their partner also carries this allele) for a deficiency of AAT1. No haplotypes of the KK342 EE264, KK342 EV264, or KK342 VV264 genotypes were observed, which is logical because as said protein is absent or at most expressed in only 15% of the population, the donor would have had to seek treatment due to illness, as previously mentioned.

Frequencies of the other EE342 EV264-mutated haplotypes accounted for 18.7% of the samples, and as for the EE342 VV264 haplotype, 0.89% of the total healthy population presented EV264 heterozygous for the mutation and one individual homozygous for said VV264 mutation. Genetic counseling would be unnecessary since this allele has less clinical relevance. No EK342 VV264, KK342 EV264, or KK342 VV264 haplotypes were observed.

### 3.5. Estimation of the AAT1 Pi Genotype in the Murcian Population

Analyzing the allelic, genotypic, and haplotype frequencies of the E342K and E264V AAT1 mutations made it possible for us to estimate the Pi genotypes of AAT1 mutations in the population of the Region of Murcia, as shown in Table 3 (lower position). The frequency of the Pi*MZ genotype was slightly lower in men than in women (2.6% vs. 4.4%). Similarly, frequencies of the Pi*SS and Pi*SZ genotypes were slightly higher in men than women and slightly lower compared to the Pi*MZ genotype (2.6% vs. 0.89%, respectively).

## 4. Discussion

This study addressed the genotypic frequencies of mutations associated with AAT1 deficiency in unrelated bone marrow donors from the Region of Murcia donor registry in southeastern Spain due to the high risk of presenting with different pathologies and underdiagnosis in the population.

First, it is interesting to note that the work presented in this report is the first of its kind carried out in the Region of Murcia. There were no previous studies regarding the diversity, polymorphism, and haplotypic associations of SERPINE1 gene mutations in the healthy population.

Pi genotype polymorphism has been determined since its discovery by isoelectric focusing. It is a tedious technique, often replaced with gene determination, which is much more versatile and sensitive than previous classical serological techniques [31].

Genetically and populationally, AAT1 deficiency is an autosomal recessive disorder in which the most commonly deficient allele, Pi*Z, is more prevalent in northern and western European countries, being rarer in southern European, Asian, and Asian populations. However, Pi*S allelic variants are more prevalent in southern European countries [32,33]. Data provided by several studies indicate that, in Spain, frequencies of Pi*S and Pi*Z would be 104 and 17 per thousand, respectively. If these results are extrapolated to the entire population of Spain, there could be a total of 9,173,181 individuals with a heterozygous phenotype for these alleles. Of the total deficient phenotypes, 80% would be Pi*MS, 13% would be Pi*MZ, 4.7% would be Pi*SS, 1.6% would be Pi*SZ, and 0.1% would be Pi*ZZ. According to penetrance approximations for the Pi*ZZ phenotype, it is estimated that 2526 adults would have COPD and 4030 subjects would have chronic liver disease [3,32].

As for Europe, the highest prevalence of Pi*ZZ genotypes (1:1500–1:2000) is found in the Baltic republics south of the Scandinavian peninsula (Norway and Sweden) and Denmark. Although declining, they remain high (1:2500–1:4000) in Belarus, Ukraine, Poland, Germany, the Netherlands, France, the British Islands, and the Iberian Peninsula. Their prevalence gradually decreases from west to east and in the outermost regions of northern and southern Europe [34].

In this sense, AAT1D is rare, but it is one of the hereditary conditions most commonly underdiagnosed worldwide [34]. The disease diagnosis is usually delayed between 7 and 10 years, with patients needing several medical consultations before reaching the correct diagnosis [34]. The causes of ignorance about AAT1D that contribute to its underdiagnosis may be as follows: doctors’ unawareness of the lung and liver diseases that can be caused by AAT1D [35]; clinical variability in the presentation of AAT1 deficiency, since up to a third of Pi*ZZ patients and two-thirds of Pi*SZ patients do not present with any clinical symptoms; medical misdiagnosis in patients classified as having COPD or liver cirrhosis associated with tobacco or alcohol and not with AAT1D [17].

Let us suppose a comparison is made between genotypic frequencies in the Spanish and Murcian populations, using data from previously published articles [3]. In that case, the data obtained in our study are very similar and confirm alongside other studies that the Pi*S allelic variant is highly prevalent in southern European countries, including Spain. It should be clarified that only the healthy population was considered in the Murcian population in our study, since they were donors without previous pathologies. In contrast, data from the whole of Spain considered the entire population since, as mentioned, ours is the first study carried out exclusively on a healthy population.

These frequencies are more similar to those found in neighboring populations such as the Italian population (where it is slightly higher) and the French population (slightly lower) [36], which is logical due to the geographic proximity and the less dispersed gene flow typical of Mediterranean populations. However, the proportion of frequencies for these mutations in Portugal [34] compared to their total in Spain and the region of Murcia, in particular, is surprising. Portugal, possibly the closest in terms of geographical location to Spain, as part of the Iberian Peninsula, shows a similarity and genetic proximity in our studies on carried HLA polymorphism. In particular, Murcia, the rest of Spain, and Portugal form almost a unit in the dendrograms and correspondence analyses of our previous studies concerning the variances of the HLA allele frequencies and even of mutations of other genes, such as HFE, responsible for hemochromatosis [37,38]. The slight divergence regarding the SERPINE1 gene in Portugal, compared to Spain, may be due to the small number of Portuguese studies carried out on this specific gene and should be studied in the future.

Regarding the individual frequencies of genotypes, the analyses performed in this study did not show the existence of any subject presenting a PI*ZZ genotype since the samples we used were those of healthy people, and in the case of PI*ZZ, the donor would have had to seek treatment due to illness and the genotype would have been detected quickly. We found five individuals carrying the PI*MZ genotype who may present an increased risk of suffering from pulmonary emphysema and liver disease, obviously more aggravated if they are habitual smokers [3,34].

Analogously, the PI*SZ genotype (see Table 3) appeared in two healthy individuals who could develop or perhaps already had a slight onset of emphysema (20–50%) and/or slight liver disease, which is not a negligible fact. As defined by some authors, translated [36], “this AAT1 deficiency is not such a rare disease, but rather a rarely diagnosed disease”.

These results show the importance of the genetic factor in the development of diseases related to AAT1 deficiency, specifically COPD. A surprising fact is the high presence (18.75%) of the Pi*S allelic variant in the healthy population unaware of carrying this mutation. If a subject who carries this mutation smokes, this environmental factor probably contributes to their developing the disease and suffering a more severe pathology. Given this situation, it would be interesting to carry out a public health study on the Region of Murcia or at the national level to allow the early diagnosis of heterozygous subjects carrying one of the most prevalent mutations to avoid the most harmful effects of the disease. In addition, it is necessary for carriers to eradicate tobacco use as soon as possible and start treatment earlier. Therefore, this high prevalence of the Pi*S allelic variant and the presence of the Pi*MZ and Pi*SZ genotypes in the healthy population of the Region of Murcia should be known by pulmonology services and health and safety authorities in the region, in case they consider screening the smoking population of the Region of Murcia.

Thanks to this study, it was possible to estimate, for the first time, the genotypic frequencies of the mutations associated with AAT1 deficiency in the Region of Murcia. On the other hand, our study has several limitations, such as the sample size used; therefore, the conclusions of our work should be validated in larger cohorts. In addition, the design of a single-center study may limit extending our general conclusions.

## 5. Conclusions

In conclusion, the frequencies of Pi mutations in a healthy population from the Region of Murcia were not remarkably different from those of the few studies reported in Spain. The presence of allelic variants, mainly Pi*S, represents a risk factor for the health of smokers in the development of COPD since it is unknown and is not screened for in the healthy population of the region. The genotype and haplotype frequencies of the healthy Murcian population follow the usual pattern of the Spanish population. There is a need to continue researching AAT1 and its relationship with specific diseases to eradicate its underdiagnosis worldwide. This high prevalence of the Pi*S allelic variant and pathological genotypes such as Pi*MZ and Pi*SZ in the healthy population of the Region of Murcia should be known by the Region’s health authorities in case they consider carrying out screening in the smoking population of the Region of Murcia.

## Figures and Tables

**Table 1:** Different forms of genetic variation in the SERPINE1 gene analyzed in this study.

Variants	Exon	Mutation	Variation	Reference
S	IV	GAA → GTG	Glu^264^ → Val^264^ (E264V)	[27]
Z	V	GAG → AAG	Glu^342^ → Lys^342^ (E342K)	[28]

**Table 2:** Frequency of alleles (upper section) and genotypes (E342K and E264V) of SERPINE1 gene in the healthy population.

SERPINE1 Gene	Total N = 112; 2n = 224 N (%)	95% CI (%)
**Allele frequencies/variation**		
GAG/E^342^ (wild-type)	217 (96.8%)	95.9–98.7
AAG/K^342^ (mutant)	7 (3.12%)	1.41–3.99
GAA/E^264^ (wild-type)	199 (88.8%)	75.6–89.6
GTG/V^264^ (mutant)	25 (11.1%)	8.81–12.41
**Genotype frequencies**		
EE^342^	105 (93.75%)	92.8–95.2
EK^342^	7 (6.25%)	4.0–8.8
KK^342^	0 (0.0%)	0
EE^264^	88 (78.57%)	70.3–81.8
EV^264^	23 (20.53%)	18.7–23.6
VV^264^	1 (0.89%)	0.4–2.79

E—glutamic acid; K—Lysine; V—Valine; CI—Confidence Interval.

**Table 3:** Haplotype frequencies of SERPINE1 in the healthy population.

Haplotype Frequencies of SERPINE1	Total N = 112; 2n = 224 (%) N (%)	95% CI (%)
EE^342^ EE^264^	83 (74.10%)	71.7–81.1
EK^342^ EE^264^	5 (4.46%)	4.0–5.9
KK^342^ EE^264^	0 (0.0%)	0
EE^342^ EV^264^	21 (18.75%)	17.8–22.1
EE^342^ VV^264^	1 (0.89%)	0.81–1.1
EK^342^ EV^264^	2 (1.78%)	1.7–2.1
EK^342^ VV^264^	0 (0.0%)	0
KK^342^ EV^264^	0 (0.0%)	0
KK^342^ VV^264^	0 (0.0%)	0
Pi* Genotype		
Pi*MM	83 (74.10%)	71.7–84.1
Pi*MZ	5 (4.46%)	3.9–5.99
Pi*ZZ	0 (0.0%)	0
Pi*MS	21 (18.75%)	17.8–22.1
Pi*SS	1 (0.89%)	0.71–2.11
Pi*SZ	2 (1.78%)	1.07–2.15

E—glutamic acid; K—Lysine; V—valine; CI—confidence interval.

## Data Availability

Not applicable.
